# Direct Electrochemistry of Horseradish Peroxidase‐Gold Nanoparticles Conjugate

**DOI:** 10.3390/s90200881

**Published:** 2009-02-10

**Authors:** Gautham Kumar Ahirwal, Chanchal K. Mitra

**Affiliations:** Department of Biochemistry, School of Life Sciences, University of Hyderabad, Hyderabad, Andhra Pradesh, India 500 046

**Keywords:** Gold nanoparticles, glutathione, lipoic acid, horseradish peroxidase, direct electron transfer

## Abstract

We have studied the direct electrochemistry of horseradish peroxidase (HRP) coupled to gold nanoparticles (AuNP) using electrochemical techniques, which provide some insight in the application of biosensors as tools for diagnostics because HRP is widely used in clinical diagnostics kits. AuNP capped with (i) glutathione and (ii) lipoic acid was covalently linked to HRP. The immobilized HRP/AuNP conjugate showed characteristic redox peaks at a gold electrode. It displayed good electrocatalytic response to the reduction of H_2_O_2_, with good sensitivity and without any electron mediator. The covalent linking of HRP and AuNP did not affect the activity of the enzyme significantly. The response of the electrode towards the different concentrations of H_2_O_2_ showed the characteristics of Michaelis Menten enzyme kinetics with an optimum pH between 7.0 to 8.0. The preparation of the sensor involves single layer of enzyme, which can be carried out efficiently and is also highly reproducible when compared to other systems involving the layer-by-layer assembly, adsorption or encapsulation of the enzyme. The immobilized AuNP-HRP can be used for immunosensor applications.

## 1. Introduction

In recent years, research on nanomaterials has increased tremendously because of their importance as potential building blocks for a variety of nanoscale biomedical, bioanalytical, bioseparation and bioimaging applications [1-5]. The utilities of nanoparticles (NP) strongly depend upon their physiochemical characteristics and their interaction with various surface moieties. Gold nanoparticles stand apart from other nanoparticles and quantum dots because of their biocompatibility. The presence of gold nanoparticles (AuNP) provides more freedom in the orientation of the immobilized protein molecule thus permitting proteins to orient for direct electron transfer. Gold nanoparticles have been used for coupling to proteins [6], DNA [7-9] and RNA in various applications like immunoassays [10-11], detection of analytes [12-15], and toxic compounds [16], for nuclear targeting [17], as carrier agents [18], and also as enzymes for biosensors [19-23].

Generally, gold nanoparticles are synthesized by the reduction of an aurate salt with reducing agents, such as sodium borohydride (NaBH_4_), thiocyanate, phosphorus, citrate and ascorbate. The synthesized nanoparticles are of nanometer size, with colors varying from yellow‐orange to red‐purple to blue‐green. Nanoparticles have to be surface modified to make them stable and compatible for preparation of bioconjugate and some functional groups, such as cyano (‐CN), thiol (‐SH) and amino (‐NH_2_) groups, are known to have high affinity for gold and the molecules having such functional groups can be used as capping agents for gold nanoparticles.

Different methods have been developed till now for the synthesis and protection of the gold nanoparticles apart from the classical methods [24-26], using tryptophan [27], amines [28-29] cinnamic acid [30], polypeptides stabilized [31-32], ethylene glycol protected [33], glutathione [34], lipoic acid-Poly (γ‐benzyl‐L‐glutamate) [35].

**Scheme 1.**
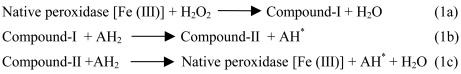
The reactions in the enzymatic catalytic cycle of HRP

The capped gold nanoparticles are used for coupling of biomolecules, and a suitable enzyme, which can be used for this coupling is horseradish peroxidase (HRP). HRP has been used for the detection purpose because of the small size and high stability to the chemical modifications. Peroxidases are enzymes of the EC 1.11.X.X class, which are defined as oxidoreductases that use hydroperoxides as electron acceptor. It has been found that peroxidases such as plant peroxidases, cytochrome c peroxidase, chloroperoxidase, lactoperoxidase etc, are heme proteins with a common catalytic cycle [36] (Scheme 1). HRP is a globular glycoprotein with a mass of 42 kDa, of which the protein moiety is approximately 34 kDa, the rest of the molecular weight being accounted for by the prosthetic group (b-type heme), two calcium ions and some surface bound glycans.

The first reaction (1a) involves the two-electron oxidation of the ferriheme prosthetic group of the native peroxidase by H_2_O_2_ (or organic hydroperoxides). This reaction results in the formation of an intermediate, compound-I (oxidation state +5), consisting of oxyferryl iron (Fe (IV) 0=O) and a porphyrin π cation radical. In the next reaction (1b), compound-I loses one oxidizing equivalent upon one-electron reduction by the first electron donor AH_2_ and forms compound-II (oxidation state +4). The later in turn accepts an additional electron from the second donor molecule AH_2_ in the third step (lc), whereby the enzyme is returned to its native resting state, ferriperoxidase.

**Figure 1.**
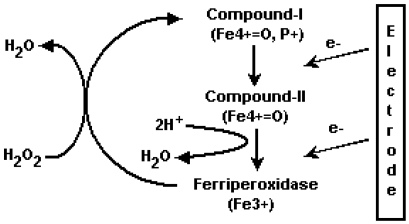
Mechanism of the direct bioelectrocatalytic reduction of hydrogen peroxidase at peroxidase-modified electrodes. P^+^ is a cation radical localized on the porphyrin ring or polypeptide chain.

Direct electrochemistry has been observed for the adsorbed peroxidase. There was a registered reduction in the current and peroxide concentration that was observed in gold [37], graphite [38-39] and platinum [40]. The electrode current was found due to an electrochemical reduction of compound‐I and compound‐II as schematically presented in Figure 1 below. In this work, we have explored the electrochemistry of covalently coupled enzyme.

The direct assembly of monolayer HRP onto the metal surface often results in the denaturation and significant loss enzyme activity. Earlier workers have reported the use of AuNP modified electrodes for immobilizing the enzyme, by using layer by layer enzyme assembly [41-45], entrapping the enzyme in a silica sol gel [46-47], by electrostatic interactions [48], and by using a carbon paste electrode [49]. To the best of our knowledge no process which involves direct covalent linking of the enzyme to the AuNP has been reported. In this work we describe a new approach for the development of sensors by directly linking the HRP to the AuNP by using the crosslinking reagent carbodiimide, which forms an amide bond between the carboxylic group of capped AuNP and amino groups present in the HRP. In this way, the enzyme is freely available in the solution while attached to the AuNP with a linker. This approach allows the substrate to approach the enzyme without encountering steric hindrance form the AuNP. This process is a simple and stable method for enzyme immobilization. The HRP was linked to both the glutathione and lipoic acid capped AuNP and immobilized onto the gold electrodes to study the electrochemical properties.

## 2. Results and Discussion

### 2.1. UV-Visible spectroscopy studies

**Figure 2.**
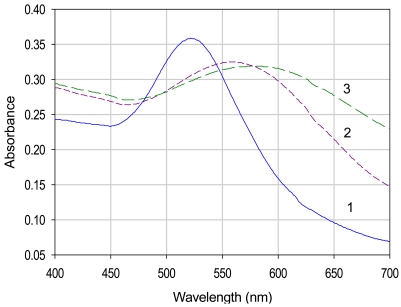
UV‐Visible spectrum of (1) gold nanoparticles (AuNP), (2) glutathione capped AuNP and (3) lipoic acid capped AuNP.

**Figure 3.**
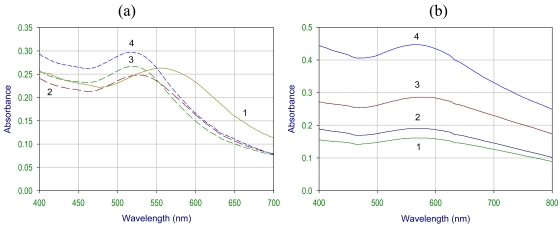
UV-Visible spectrum of (a) glutathione capped Au‐NP, and (b) lipoic acid capped Au‐NP in pH (1) 5.0, (2) 5.5, (3) 6.0, (4) 7.0 solutions.

The gold nanoparticles synthesized by borohydride reduction of aurate salt are relatively monodisperse in colloidal solution, which is confirmed by a single peak in the absorbance spectra (Figure 3). The λ_max_ was observed at around 530 nm. The dynamic laser scattering experiments suggested that the mean size of the AuNP is in the range of 20‐30 nm and after coupling to the enzyme, the approximate size was 50‐60 nm. In next step, which involved the protection of nanoparticles for stability, we have used two different capping or protecting agents i.e., glutathione and lipoic acid. As shown in Figure 2, the peak is shifted towards the higher wavelength after capping with glutathione and lipoic acid and the λ_max_ was observed around 540-580 nm for glutathione capped and 560-620 nm for lipoic acid capped gold nanoparticles. The change in the color of the colloid was also seen before and after capping. The color of the gold colloid changed from wine red to blue for glutathione and dark blue for lipoic acid capped nanoparticles.

The λ_max_ shift in the absorbance spectra was mainly due to the surface modification of the gold nanoparticles. The surface plasmon resonance, the major cause for the absorption, is affected by surface modification with covalent coupling. It may also be due to the increase in their size, which is due to the protective coating of the organic molecule. This precipitation of nanoparticles was seen in both the cases of GSH and LPA capped Au-NP. The precipitation is pH dependent. After borohydride reduction of aurate salt the pH of the solution increased (towards the alkaline side) and due to the negatively charged surface of the nanoparticles (because of the Cl^-^ ions adsorbed on the surface), the precipitation of the colloid was prevented after capping. The colloid behaved more likely as a hydrophilic macromolecule (like a protein). But as glutathione or lipoic acid is added to the colloidal solution there is a decrease in the pH (towards the acidic side). In the case of glutathione capped gold nanoparticles as shown in Figure 3a, at pH 5.0 the spectra has shifted towards the higher wavelength and as the pH is increased the shift was seen towards the lower wavelength of the spectra. Glutathione is a tripeptide (glutamic acid, cysteine and glycine) and has many binding points for the gold nanoparticles. There are two carboxylic groups, one thiol group and three amino groups in glutathione. The thiol group is involved in the attachment with the AuNP. The covalent coupling can be extended either via the carboxylic or the amino groups (of glutathione/ lipoic acid).

In the case of lipoic acid capped nanoparticles, the disulfides are reduced by borohydride to two thiol groups (–S–S– → –SH + –SH), which are involved in the binding of lipoic acid to gold nanoparticles. In this type of capping, pH dependent precipitation of nanoparticles was also observed. As shown in Figure 3b, at pH 7.0 the NPs are well dispersed and with the increase in the acidity of the solution, the broadening of peak was seen, which indicates the precipitation of NP in the solution. This property of capped NP precipitation was utilized for the separation of NP from the unreacted organic molecule.

### 2.2. Fourier transform infrared spectroscopy studies of Au-NPs

**Figure 4.**
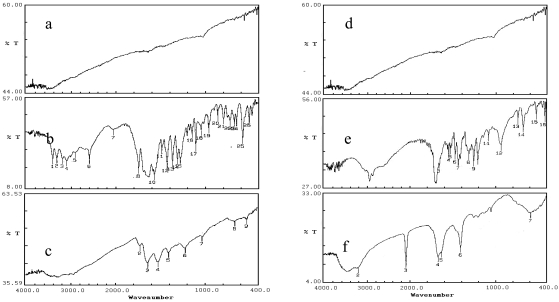
FT‐IR (JASCO FT/IR‐5300) spectrum in KBr of (a) gold nanoparticles, (b) glutathione powder and (c) gold nanoparticles capped with glutathione and (d) gold nanoparticles, (e) lipoic acid powder and (f) gold nanoparticles capped with lipoic acid.

FTIR measurements were carried out on neat gold nanoparticles (Figures 4a and 4d), glutathione (Figure 4b), lipoic acid (Figure 4e) and gold nanoparticles capped with glutathione (Figure 4c) and lipoic acid (Figure 4f).

**Figure 5.**
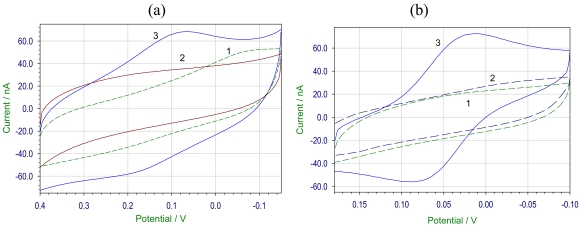
Cyclic voltammogram of HRP coupled to (a) glutathione capped AuNP, (b) lipoic acid capped AuNP. Using gold electrode, 10.2 μM H_2_O_2_, 20 mV/s scan rate, Ag/AgCl reference electrode. Plot (1) is blank electrode, (2) AuNP modified electrode and (3) is HRP coupled AuNP modified electrode

We note that after coupling to the gold nanoparticles, a number of peaks present in the free molecules (Figure 4b: peaks at 549 cm^-1^, probably S-S stretch, and 2,525 cm^-1^, probably S-H stretch; Figure 4e: 671 cm^-1^ and 518 cm^-1^, probably S-S stretch) practically disappear (Figures 4c, 4f). Characteristic frequencies for the peptide bond are not significantly affected (Figure 4b: 3,128 cm^-1^ and 3,032 cm^-1^; Figure 4c: 3,271 cm^-1^; Figure 4e: 1,657 cm^-1^ and 1,541 cm^-1^; Figure 5f: 1,666 cm^-1^ and 1,626 cm^-1^), as expected. We suggest that the vibrations are quenched or shielded by the gold nanoparticles and the energy is transferred to the internal modes of the nanoparticles. One of the reasons may be the molecules which are attached to the nanoparticles on the side of IR source are getting absorbed and the vibrational energy is not transmitted to the detector, whereas the molecules on the other side of the nanoparticles are getting transmitted but vibrational energy is not sufficient to be detected. The S-S stretch vibration disappears completely in glutathione after coupling and S-H stretch vibration disappears completely in lipoic acid after coupling. A few vibrational modes survive and can still be seen in the IR spectrum of the coupled gold nanoparticles.

### 2.3. Electrochemical studies of HRP coupled to Au-NPs

In the next step the capped nanoparticles were used for studying the binding properties of the NP to the proteins. For this study we have used horseradish peroxidase (HRP) and used cyclic voltammetry to study the electrochemistry.

HRP was coupled to both glutathione and lipoic acid capped gold nanoparticles using carbodiimide coupling. There was no change in the enzyme activity of HRP after coupling to gold nanoparticles and the coupled NP was used for the cyclic voltammetric studies.

#### 2.3.1. Electrochemical studies of HRP coupled capped AuNPs

The cyclic voltammogram of 10.2 μM H_2_O_2_ in pH 7.2 phosphate buffer at gold modified electrode with HRP labeled gold nanoparticles showed a couple of redox peaks [HRP coupled to glutathione capped AuNP, Figure 5(a), and lipoic acid capped AuNP, Figure 5(b)] at the potentials of Epc = 0.064 V; Epa = 0.17 V and Epc = 0.011 V; Epa = 0.089 V respectively. The cathodic and anodic peaks results from the redox process at the electrode modified by HRP-AuNP conjugate, when compared to the blank cyclic voltammogram where no redox peaks were observed. The reduction of H_2_O_2_ was better at the electrode modified with HRP labeled to lipoic acid capped AuNP as the peak separation value ΔEp was ∼39 mV. The direct electron transfer reaction between the cofactor of HRP and the electrode surface was due to the favored orientation of HRP molecule or gold NP acting as the conducting channels for the electron transfer. Other workers have reported significantly different potentials (Epa ∼ 160 mV and Epc ∼190 mV) using glassy carbon (which often shows high overpotential to electron transfer) as the base electrode [44]. Our results are closer to 0 mV suggesting a more efficient electron transfer.

**Figure 6.**
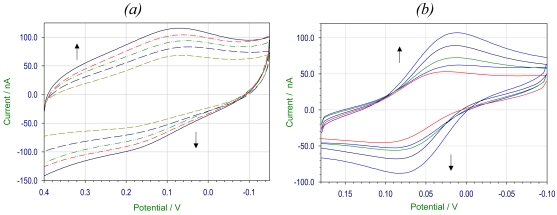
Cyclic voltammogram of HRP coupled to (a) glutathione capped AuNP, (b) lipoic acid capped AuNP at scan rates of 5, 10, 20, 40 and 80 mV/s.

Figure 6 shows the cyclic voltammogram of immobilized HRP‐AuNP at various scan rates. We have observed that there is a linear increase of cathodic and anodic peak current values with the increase of scan rate. The peak potentials shift slightly with the change in the scan rate.

#### 2.3.2. Electrochemical studies of HRP coupled to AuNPs at different concentrations of H_2_O_2_ and pH

**Figure 7.**
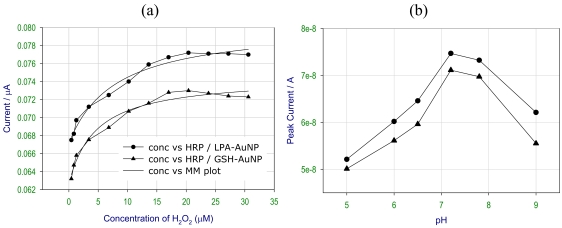
Dependence of the peak current values obtained from cyclic voltammogram of HRP coupled to lipoic acid capped (-●-) measured at the potential of 0.06 V and glutathione capped AuNP (-▲-) measured at a potential 0.01 V using (a) different concentrations of H_2_O_2_ and at (b) different pH.

The electrochemical studies of HRP-AuNP conjugate were carried out using different concentrations of H_2_O_2_, as shown in Figure 7a. There was an increase in the peak current value with the increase in the peroxide concentration and at higher concentration a plateau was obtained. A hyperbolic plot, mimicking Michaelis Menten (MM) kinetics have been fitted to the experimental points for a better visual clarity. The MM plot obtained for both the HRP coupled to glutathione and lipoic capped gold nanoparticles matches with the normal plot reasonably well. The optimum concentration for good electrochemical response was observed in between 6.8‐20.4μM concentration of H_2_O_2_. At higher concentrations of peroxide, HRP was transformed into the inactive form, which resulted in the no change in the response such behavior is characteristic response to the enzyme substrate kinetics.

Figure 7b shows the direct electron transfer of immobilized HRP activity at various pH. A pH range of 7.0-8.0 was optimal for the good electroactivity of the enzyme. At lower pH values the activity of HRP decreased due to the denaturation of the enzyme and at a higher pH the enzyme converted into a completely inactive form. These results were consistent with the known biochemical characteristics of the enzyme. Therefore the observed pH dependence was very similar for both glutathione capped and lipoic acid capped AuNPs.

### 2.4. Activity studies

The activity of immobilized enzyme (heterogeneous catalysis) is not directly comparable with soluble enzymes and hence we have compared the activity of the enzyme (units/mg of protein) after removing the thiol linkage. The resulting activities (compared to the soluble enzyme used as standard) are within the limits of the experimental errors. This suggests that practically all the enzyme retained their biochemical activity.

## 3. Experimental Section

### 3.1. General

HRP (EC 1.11.1.7. RZ ∼3, >250u/mg) and *N*-Ethyl-*N*′-(3-dimethylaminopropyl) carbodiimide hydrochloride (EDC) were purchased from Sigma, AuCl_4_.4H_2_O (Au% > 49%). All other chemicals were of the analytical grade and were used without further purification. All the solutions were prepared using double distilled water. The absorption spectrum of the samples was recorded with an UV‐1601 spectrophotometer (Shimadzu). FTIR characteristics of the samples were collected on a Jasco type 5300 FT-IR spectrometer using KBr mulls. Electrochemical measurements were performed using a CH Instruments 660A with a three electrode system comprising of platinum auxiliary electrode, a Ag/AgCl reference electrode and gold electrode as working electrode. The 1:1 ratio of 10 mM phosphate buffer (PB) and 100 mM KCl was used as electrolyte for all the measurements.

### 3.2. Preparation of gold nanoparticles (AuNP)

**Scheme 2.**
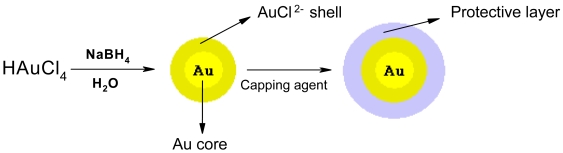
Schematic representation of the synthesis and stabilization of AuNP.

All the glassware used for the preparation were cleaned and soaked in freshly prepared HNO_3_/HCl mixture, and then they were rinsed thoroughly in distilled water and dried in air. In a typical preparation of gold colloid, glutathione and lipoic acid were used for capping of gold nanoparticles. The gold colloid was prepared by dissolving aurate salt (12 mg) in double distilled water (100 mL) and then freshly prepared NaBH_4_ solution (2.5 mg/mL, 400 μL) was added dropwise with stirring. The solution was kept for stirring for around 5 minutes (Scheme 2).

#### 3.2.1. Preparation of glutathione capped AuNP

To the gold colloid solution (25 mL), glutathione (20 mg) was added and the mixture was stirred for 10‐15 mins. After the stirring was completed the mixture was centrifuged at 4,500 rpm to separate the capped AuNPs. The pellet obtained was resuspended in 1 mL of phosphate buffer (pH 7).

#### 3.2.2. Preparation of lipoic acid capped AuNP

Lipoic acid solution (15 mg/mL) was prepared by dissolving 15 mg of Lipoic acid in 1 mL of ethanol-water (1:1 ratio). The lipoic acid solution (1mL) was then added to the gold colloid solution (25 mL) while stirring and after 10‐15 minutes of stirring the resulting capped nanoparticles was separated by centrifuging at 4,500 rpm. The pellet obtained was resuspended in 1 mL of phosphate buffer (pH 7).

### 3.3. Preparation of AuNP –HRP conjugate

**Scheme 3.**
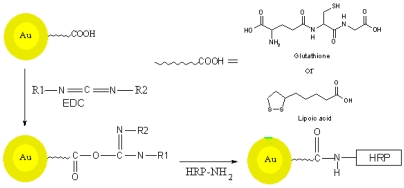
Schematic representation of the covalent coupling of HRP to capped AuNP.

The covalent coupling process using EDC generally involves two steps, one is activation of the carboxylic acid group, and this step is carried out at slightly acidic pH between 4.5-5.0. The second step involves the amide bond formation between the activated carboxylic groups of one species with amino group of the other and can be carried out at basic pH (as shown in Scheme 3). The property of pH dependent precipitation of AuNP was utilized for the crosslinking of HRP and AuNP, at acidic pH the free carboxylic group is activated by EDC.

#### 3.3.1. Activation of carboxylic groups in AuNP

Both the glutathione and lipoic acid capped AuNP was used as separate batches for the activation. The capped AuNP was added to pH 5.0 MES buffer (3 mL) and 58 mM EDC (300 μL) was added and the reaction mixture was kept at 4 ^°^C for 1.5 hrs on a rocker. After the reaction has completed the mixture was centrifuged to separate the activated AuNP from the unreacted EDC. The pellet obtained was than washed with water twice and resuspended in the phosphate buffer.

#### 3.3.2. Covalent linking of HRP with AuNP

To the activated AuNP HRP (3mg/mL in water, 150 μL) was added and the reaction mixture was kept at 4^°^C overnight. The resulting HRP-AuNP conjugate was separated by centrifugation and dissolving the pellet in phosphate buffer. The enzyme assay for peroxidase was performed using pyrogallol. The modified electrode was prepared by putting HRP-AuNP conjugate (5 μL) onto the cleaned and polished gold electrode and allowing it to dry at room temperature. After drying the electrode was rinsed with buffer and used for the electrochemical measurements.

## 4. Conclusions

Glutathione and lipoic acid capped gold nanoparticles were prepared by borohydride reduction, which were directly used for covalently attaching the HRP and the resulting AuNP-HRP bioconjugate was immobilized onto the gold electrode by direct adsorption. The cyclic voltammetry measurement showed good electrochemical response for both HRP coupled to glutathione and lipoic acid capped Au‐NP. The peak potential was at Epc = 0.064 V; Epa = 0.17 V and Epc = 0.011 V; Epa = 0.089 V for glutathione and lipoic acid capped gold colloid. The lipoic acid capped Au‐NP modified electrode showed a better electrochemical response when compared to the glutathione capped one. The optimal activity of HRP labeled AuNP was between pH 7.0-8.0. This study shows that the approach of covalent linking of the enzyme to the nanoparticles needs less time, forms a more stable attachment (no leaching). Coupled enzyme has full biochemical activity with a spacer arm, which gives a possibility of higher efficiency of electron transport.

A large number of clinical diagnostic tests depends on or uses HRP as a reported enzyme and uses a color reaction in which a suitable dye is used as an electron acceptor. Also, conventional ELISA tests depend exclusively on HRP to report the presence of selected antibodies. We propose that the HRP electrode can be successfully used as an electrochemical tool instead of a colorimetric assay, as this is a faster, more accurate and less prone to noise. The protocol outlined in this experiment can be used for any common enzyme with very little modifications and therefore offers the possibility of developing arrays of sensors using this covalent coupling technique.
